# Assembly, growth and conductive properties of tellurium nanorods produced by *Rhodococcus aetherivorans* BCP1

**DOI:** 10.1038/s41598-018-22320-x

**Published:** 2018-03-02

**Authors:** Alessandro Presentato, Elena Piacenza, Ali Darbandi, Max Anikovskiy, Martina Cappelletti, Davide Zannoni, Raymond J. Turner

**Affiliations:** 1 Microbial Biochemistry Laboratory, Department of Biological Sciences, University of Calgary, 2500 University Dr. NW, Calgary, AB T2N 1N4 Canada; 2 Microscopy and Imaging Facility, Cumming School of Medicine, University of Calgary, 3330 Hospital Dr. NW, Calgary, AB T2N 4N1 Canada; 3 Department of Chemistry, University of Calgary, 2500 University Dr. NW, Calgary, AB T2N 1N4 Canada; 4 Unit of General and Applied Microbiology, Department of Pharmacy and Biotechnology, University of Bologna, Via Irnerio 42, Bologna, 40126 Italy

## Abstract

Tellurite (TeO_3_^2−^) is a hazardous and toxic oxyanion for living organisms. However, several microorganisms can bioconvert TeO_3_^2−^ into the less toxic form of elemental tellurium (Te^0^). Here, *Rhodococcus aetherivorans* BCP1 resting (non-growing) cells showed the proficiency to produce tellurium-based nanoparticles (NPs) and nanorods (NRs) through the bioconversion of TeO_3_^2−^, depending on the oxyanion initial concentration and time of cellular incubation. Te-nanostructures initially appeared in the cytoplasm of BCP1 cells as spherical NPs, which, as the exposure time increased, were converted into NRs. This observation suggested the existence of an intracellular mechanism of TeNRs assembly and growth that resembled the chemical surfactant-assisted process for NRs synthesis. The TeNRs produced by the BCP1 strain showed an average length (>700 nm) almost doubled compared to those observed in other studies. Further, the biogenic TeNRs displayed a regular single-crystalline structure typically obtained for those chemically synthesized. The chemical-physical characterization of the biogenic TeNRs reflected their thermodynamic stability that is likely derived from amphiphilic biomolecules present in the organic layer surrounding the NRs. Finally, the biogenic TeNRs extract showed good electrical conductivity. Thus, these findings support the suitability of this strain as eco-friendly biocatalyst to produce high quality tellurium-based nanomaterials exploitable for technological purposes.

## Introduction

The chalcogen Tellurium (Te) is a natural rare element of the Earth crust^1^ that is defined as a metalloid due to its intermediate properties between metals and non-metals^2^. The anthropogenic misuse of Te-compounds in several areas of application (i.e., electronics, optics, production of batteries, petroleum refining and mining)^1,3–5^ has led to an increased presence of several forms of Te in the environment, namely: inorganic telluride (Te_2_), the oxyanions tellurite (TeO_3_^2−^) and tellurate (TeO_4_^2-^), and the organic dimethyl telluride (CH_3_TeCH_3_)^6^. Among these Te forms, TeO_3_^2−^ is recognized as a soluble and hazardous pollutant, which can be found highly concentrated in soils and waters near by waste discharge sites of manufacturing and processing facilities^7^. Although TeO_3_^2−^ exerts its toxicity at concentrations as low as 1 μg mL^−1^ (4 μM)^5^ towards both prokaryotes and eukaryotes^6^, over the past 30 years mainly anaerobic or facultative anaerobic bacteria were investigated for their ability to bioconvert TeO_3_^2−^ ^1,8,9^, while much less is known about the bioconversion potential of aerobic bacterial strains towards these oxyanions^10–12^. Regardless of the bacterial strain investigated, a common feature reported by several authors, is that TeO_3_^2−^ bioconverting bacteria produces black precipitates within and/or outside the cells^13,14^. Indeed, the early work of Morton and Anderson (1941) observed needle-like crystals within and outside *Corynebacterium diphtheriae* cells grown on Chocolate Tellurite agar^13^, while Tucker and colleagues (1962) reported X-Ray diffraction analysis of Te crystalline nature of the black precipitates produced by *Streptococcus fecalis* N83^11^. Recently, these Te-crystals were recognized as nanosized structures generated by microorganisms as product of metal(loid) bioconversion^8,15,16^, which can be exploited to develop eco-friendly and cost-effective methods to synthesize valuable metalloid nanomaterials^17^. Indeed, the advantage of a microbial approach as compared to a synthetic procedure would be the abandonment of toxic chemicals, avoiding the formation of hazardous waste, and the use of extreme system conditions (i.e., high pressure and temperature), which determine the emergence of safety concerns^17^.

In this regard, among the strictly aerobic bacterial strains suitable as cell factories for nanotechnology purposes, those belonging to the *Rhodococcus* genus have been investigated due to their environmental robustness and persistence^18^, with the characteristic of resisting harsh growth conditions^19,20^. In a previous study, we reported the ability of *Rhodococcus aetherivorans* BCP1 to cope with high concentrations of TeO_3_^2−^, as well as its proficiency to bioconvert these oxyanions into the less toxic Te^0^, generating thermodynamically stable nanostructures^21^. Here, based on our prior findings, we further explored the strain BCP1 under metabolically active, yet resting (non-growing) cells. Conditions using these cells were optimized for the biotic conversion of TeO_3_^2−^ and to enhance the chemical-physical characteristics of the biogenic Te-nanomaterial produced. We investigated key parameters such as size, shape, and crystalline nature of the Te-nanostructures biosynthesized by BCP1, and we provided evidence for the presence of amphiphilic biomolecules in the organic layer surrounding the biogenic TeNRs, which might play a crucial role directing their growth and stabilizing them. Hence, we proposed a mechanism of assembly, growth and formation of the intracellularly generated TeNRs, whose electrical properties were evaluated as proof-of-concept of the suitability of this nanomaterial for future electronic applications.

## Results and Discussion

### BCP1’s tolerance and biotic conversion of TeO_3_^2−^

**Figure 1:**
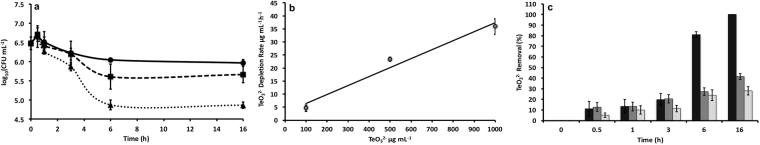
Survival curve, initial TeO_3_^2−^ depletion rate and percentage of TeO_3_^2−^ removal. (**a**) *Rhodococcus aetherivorans* BCP1 resting cells survival curve upon increased initial concentration of TeO_3_^2−^, being 100 (), 500 () or 1000 () μg mL^−1^, while in (**b**) is shown the initial depletion rate () of TeO_3_^2−^. The linear correlation () that fits the experimental data points gave an R^2^ = 0.97. In (**c**) is reported the percentage of TeO_3_^2−^ removal over the considered timeframe for each initial oxyanion concentration [100 (■), 500 () or 1000 () μg mL^−1^]. The error bars indicate the standard deviation of three biological replicates.

The exploitation of bacteria bioconverting chalcogen oxyanions^22^ is now recognized as a valuable approach to develop green-synthesis strategies to produce unique nanoscale materials^23^. In our previous study, the capability of BCP1 cells grown aerobically in the presence of TeO_3_^2−^ to biosynthesize TeNRs as product of TeO_3_^2−^ bioconversion was observed^21^. In our research exploring different physiological conditions to optimize TeNRs production, we discovered that BCP1 resting cells had a greater performance to produce extremely long TeNRs as compared to actively growing cultures^21^. Indeed, although TeO_3_^2−^ exposure caused a certain level of cell death directly proportional to the initial concentration of oxyanions (Fig. 1(a)), 100 μg mL^−1^ of TeO_3_^2−^ was bioconverted 20 h faster by BCP1 resting cells (Fig. 1(c)) as compared to the actively growing culture^21^. Similar conclusions can be drawn in the case of BCP1 resting cells exposed to 500 μg mL^−1^ of TeO_3_^2−^, even though 16 h exposure did not lead to 100% bioconversion (42 ± 3% removal). BCP1’s tolerance towards this oxyanion was further highlighted by its capability to remove 28 ± 4% (corresponding to 280 ± 40 μg mL^−1^) when exposed to 1000 μg mL^−1^ TeO_3_^2−^ over 16 h (Fig. 1(c)). In comparison, *Escherichia coli* K12 strain showed a similar TeO_3_^2−^ bioconversion trend but under anoxic conditions in the presence of the quinone electron carrier analogue lawsone^24^. The highly resistant Gram-positive aerobic bacteria *Bacillus* sp. BZ and *Salinicoccus* sp. QW6 did not bioconvert more than 100 or 125 μg mL^−1^ of TeO_3_^2−^ within 50 or 72 h exposure, respectively^10,12^. In our study, TeO_3_^2−^ removal rate was calculated after 3 h of cellular exposure to the oxyanions, as a comparable extent of live cells was detected for each experimental condition, and a linear correlation of TeO_3_^2−^ removal rate as function of the initial oxyanion concentration was observed, being the measured rates of 4.6 ± 1.3 μg mL^−1^ h^−1^ (100 μg mL^−1^), 23.4 ± 0.7 μg mL^−1^ h^−1^ (500 μg mL^−1^) and 36 ± 3.0 μg mL^−1^ h^−1^ (1000 μg mL^−1^) (Fig. 1(b)). Finally, no abiotic TeO_3_^2−^ removal was observed over the timeframe tested, as shown in the Supplementary Fig. S1.

### Electron microscopy characterization of the biogenic Te-nanomaterials

**Figure 2:**
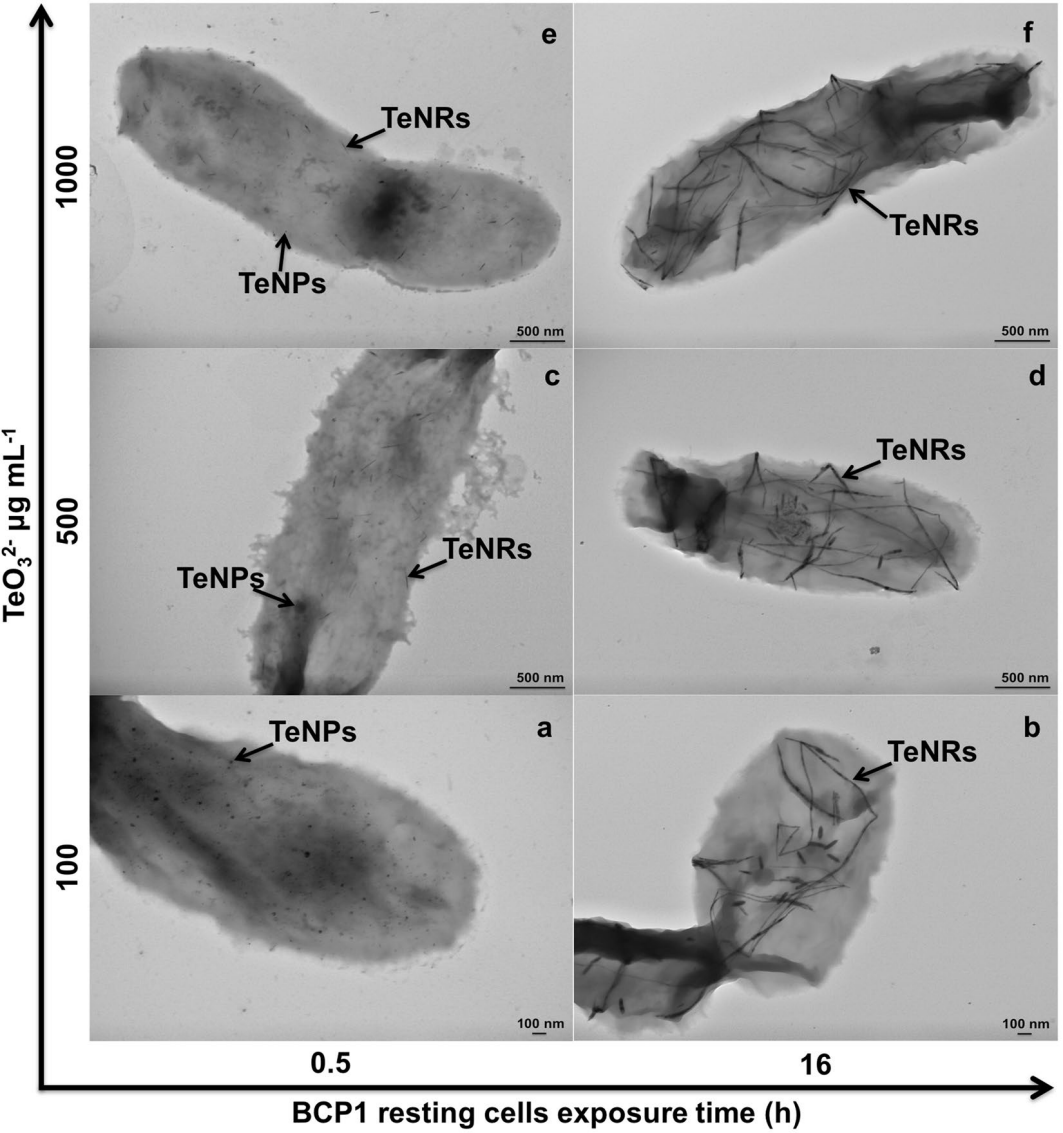
Imaging of the BCP1 strain by electron microscopy. Transmission Electron Microscopy observations of *Rhodococcus aetherivorans* BCP1 resting cells exposed to different concentrations (100, 500 and 1000 μg mL^−1^) of TeO_3_^2−^ either for 0.5 (**a**,**c** and **e**) or 16 h (**b**,**d** and **f**); TeNPs and TeNRs within the cells are indicated by black arrows.

**Figure 3:**
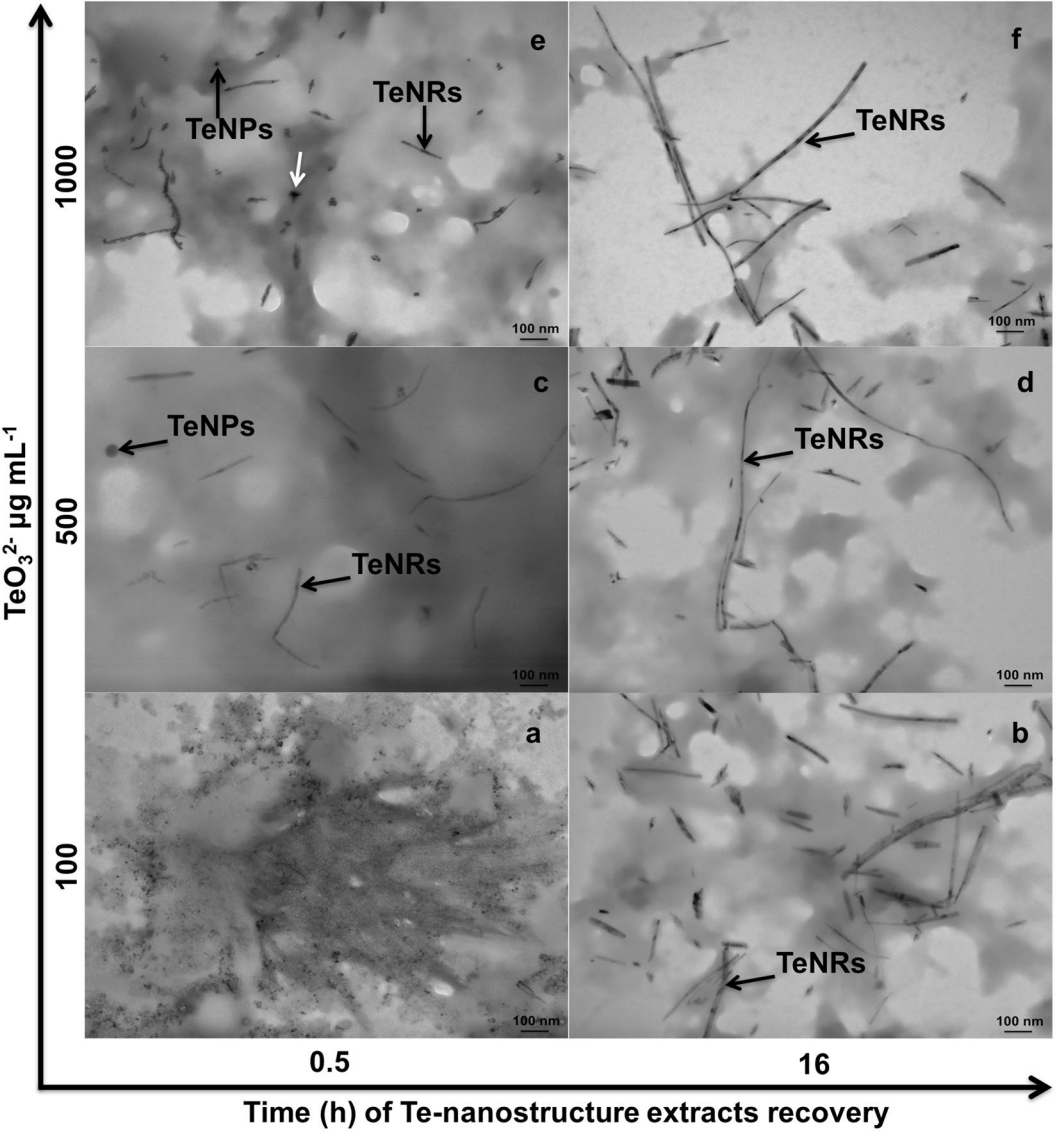
Imaging of TeNRs extracts recovered from BCP1 cells. Transmission Electron micrographs of Te-nanostructure extracts recovered after 0.5 (**a**,**c** and **e**) or 16 h (**b**,**d** and **f**) exposure of the BCP1 strain to 100, 500 and 1000 μg mL^−1^ of TeO_3_^2−^; spherical and rod-shaped Te-nanostructures, as well as shard-like NPs are indicated by black and white arrows, respectively.

BCP1’s remarkable potential in removing TeO_3_^2−^ was coupled to its proficiency to generate intracellular Te-nanostructures in the form of NPs and NRs in all experimental conditions tested (Fig. 2; Supplementary Figs S2, S3 and S4). To date, several other Gram-positive bacterial strains were recently described for their capability to generate Te-nanomaterials in the form of NRs, even if these mostly appeared either as needle-like structures or intra-/extra-cellular rosettes constituted by clustered NRs^8,25^. Conversely, the production of not aggregated intracellular TeNRs was only observed in the case of *Bacillus* sp. BZ^12^, BCP1 growing^21^ and resting cells (Fig. 2). Remarkably, TeNRs within the extracts recovered from BCP1 cells either grown^21^ or exposed to TeO_3_^2−^ still maintained their strong thermodynamic stability, even after mounting and air-drying on a carbon-coated copper grid for TEM imaging (Fig. 3; Supplementary Figs S5, S6 and S7).

Under resting cell experimental conditions, the BCP1 strain exposed to the lowest TeO_3_^2−^ concentration (100 μg mL^−1^) produced primarily TeNPs (Fig. 2(a)) at the earliest stage of incubation (0.5 h), while at higher initial TeO_3_^2−^ concentrations (i.e., 500 and 1000 μg mL^−1^) both TeNPs and TeNRs were detected within the cells (Fig. 2(c,e)). TeNPs were still observed up to 1 h after initial cellular exposure to each concentration of TeO_3_^2−^ precursor (Supplementary Figs S2b, S3b and S4b); while the production of Te-nanomaterials shifted towards 1D morphology (TeNRs) when BCP1 cells were incubated with TeO_3_^2−^ for more than 3 h (Figs 2(b,d,f)); Supplementary Fig. S2, S3 and S4). Furthermore, TEM micrographs of Te-nanostructure extracts recovered from BCP1 resting cells exposed for 0.5 h to 100 μg mL^−1^ of TeO_3_^2−^ displayed the presence of undefined electron-dense nanomaterials resembling mesoparticles (Fig. 3(a)), while defined TeNPs and TeNRs (Fig. 3(c,e) were observed as the concentration of TeO_3_^2−^ precursor increased (500 and 1000 μg mL^−1^). Shard-like NPs were also detected along with TeNRs within Te-nanostructure extracts isolated from BCP1 cells exposed for either 0.5 or 1 h to 1000 μg mL^−1^ of TeO_3_^2−^ (indicated by white arrows in Fig. 3(e)); Supplementary Fig. S7a,b). Although different morphologies of Te-nanostructures were detected, the biosynthesis was tuned towards TeNRs as the main nanomaterial product under all the experimental conditions tested (Supplementary Figs S5, S6 and S7).

**Figure 4:**
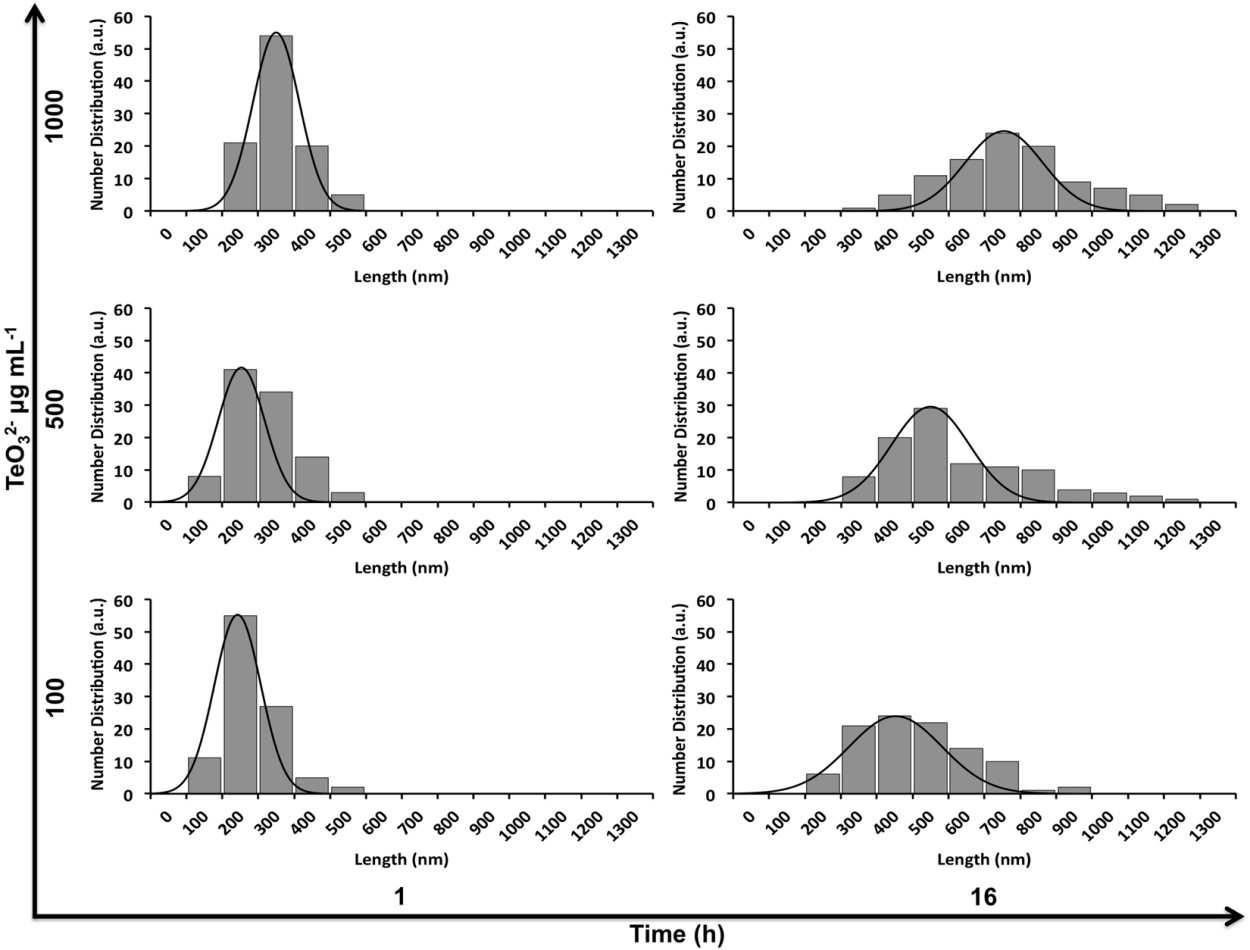
Length distribution of biogenic TeNRs. Length distribution () of TeNRs generated by *Rhodococcus aetherivorans* BCP1 resting cells exposed for either 1 or 16 h to 100, 500 and 1000 μg mL^−1^ of TeO_3_^2−^. The Gaussian fit is indicated by ().

The measurement of the average length and diameter of TeNRs was evaluated as function of both TeO_3_^2−^ exposure time and initial concentration (Fig. 4; Supplementary Fig. S8 and Tables S1 and S2). TeNRs were polydisperse in size with an average length shifting from short to very long NRs as the cellular exposure time and the initial TeO_3_^2−^ concentration increased (Supplementary Fig. S8 and Table S1). On the other hand, none of these experimental conditions influenced the measured TeNRs average diameter (Supplementary Table S2). Indeed, the growth of the nanomaterials was primarily maintained in 1D, producing very long NRs or ribbon-like structures, instead of branched nanomorphologies. The remarkable potential of BCP1 as biofactory to produce unique TeNRs is further highlighted comparing their average length (781 ± 189 nm) with that produced by actively growing cells (463 ± 147 nm)^21^ or other bacterial strains, such as *Rhodobacter capsulatus* (369 ± 131 nm)^8^, *Bacillus selenitireducens* (200 nm)^25^ and *Shewanella oneidensis* MR-1 (100–200 nm)^15^.

**Figure 5:**
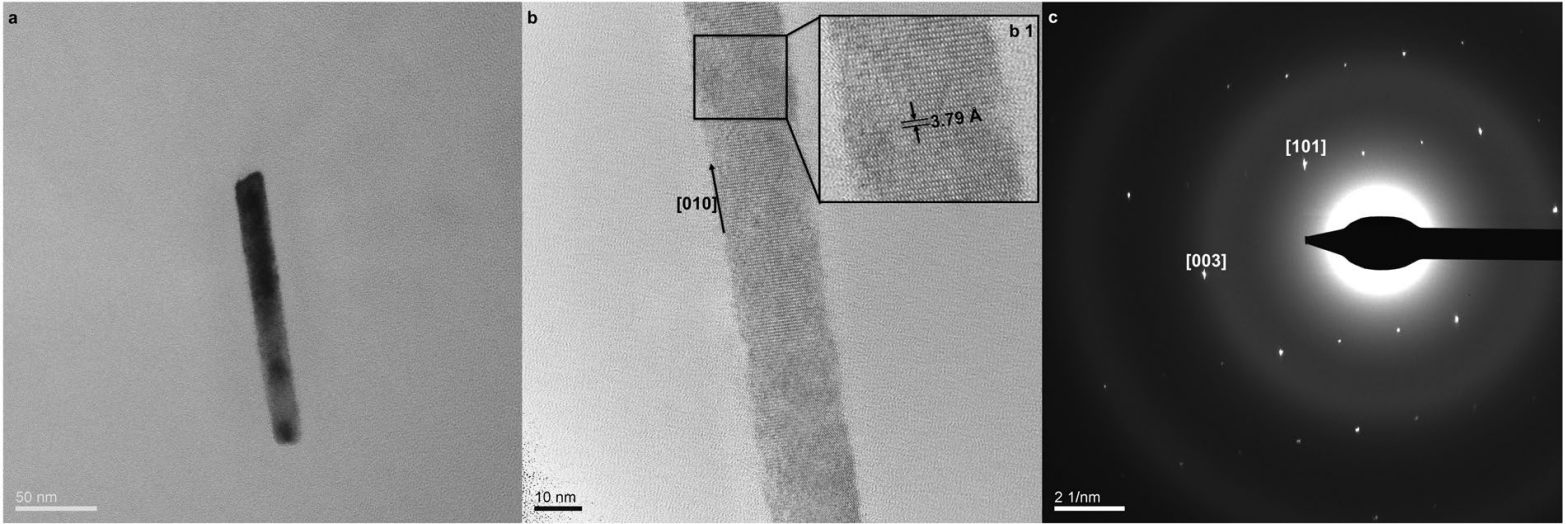
High-Resolution Transmission Electron Microscopy. (**a**) Bright-field electron micrograph of a single TeNR; (**b**) High-Resolution micrograph that highlights the [010] growth plane of TeNR crystal. The enlarged insert (b1) displays the interplanar distance of the periodic fringe spacing, while (**c**) shows the corresponding electron diffraction pattern in which the diffraction spots [101] and [003] are indexed.

Te^0^ tendency to form 1D nanostructures relies on the high thermodynamic stability of trigonal tellurium (*t*-Te), which is responsible for the anisotropic growth of Te-nanocrystallinities along one axis^26^. In this respect, the biogenic TeNRs generated byBCP1 were analyzed through HR-TEM imaging and SAED and revealed individual and regular NRs without any defects or dislocations along the longitudinal *c*-axis, indicating their uniform and single-crystalline structure (Fig. 5(b,c)). The electron diffraction (ED) patterns collected from different regions of a single TeNR confirmed the unique nature of this biogenic nanomaterial, which resembled that chemically synthesized^27^. The periodic fringe spacing of ca. 3.79 Å (Fig. 5(b)) was consistent with the established interplanar distance of ca. 3.90 Å for the separation between the [010] lattice planes of *t*-Te [space group P3_1_21(152)]^27^. Further, the TeNR ED pattern was indexed as pure *t*-Te phase with calculated lattice constants *a* = 4.38 Å and *c* = 5.83 Å (Fig. 5(c)), whose values are in line with those reported in the literature (*a* = 4.45 Å; *c* = 5.92 Å; JCPDS 36-1452).

### Mechanism of assembly and growth of the biogenic TeNRs

The nanomorphological change observed for the biogenic nanomaterial indicated a specific mechanism of assembly and growth of the NRs within BCP1 cells exposed to TeO_3_^2−^. According to the established chemical models of TeNRs synthesis^26–29^, the formation of 1D nanostructures is preceded by the generation of NPs generally featured by Te in amorphous state (*a*-Te), conferring them a high surface energy and hence thermodynamic instability. Thus, TeNPs rapidly dissolve and the available Te^0^ atoms organize themselves depositing as *t*-Te in one direction forming NRs through a ripening process^30,31^. The kinetics of this event is directly dependent on the concentration of TeO_3_^2−^ precursor supplied, resulting in a faster dissolution of TeNPs as the initial amount of oxyanion increased^30,31^. The entire process resulted to be emphasized in BCP1, as the biotic conversion of TeO_3_^2−^ occurred in the cytoplasm, leading to a large number of Te^0^ atoms confined in the small cellular volume and available to produce TeNRs. Indeed, considering that the bioconversion rate of TeO_3_^2−^ increased as function of the initial oxyanion concentration (Fig. 1(b)), BCP1 cells exposed for 0.5 h to 100 μg mL^−1^ of TeO_3_^2−^ produced only TeNPs (Fig. 2(a)), while at higher oxyanion concentrations (either 500 or 1000 μg mL^−1^) they generated both TeNPs and TeNRs at this early time point (Fig. 2(c,e)).

**Figure 6:**
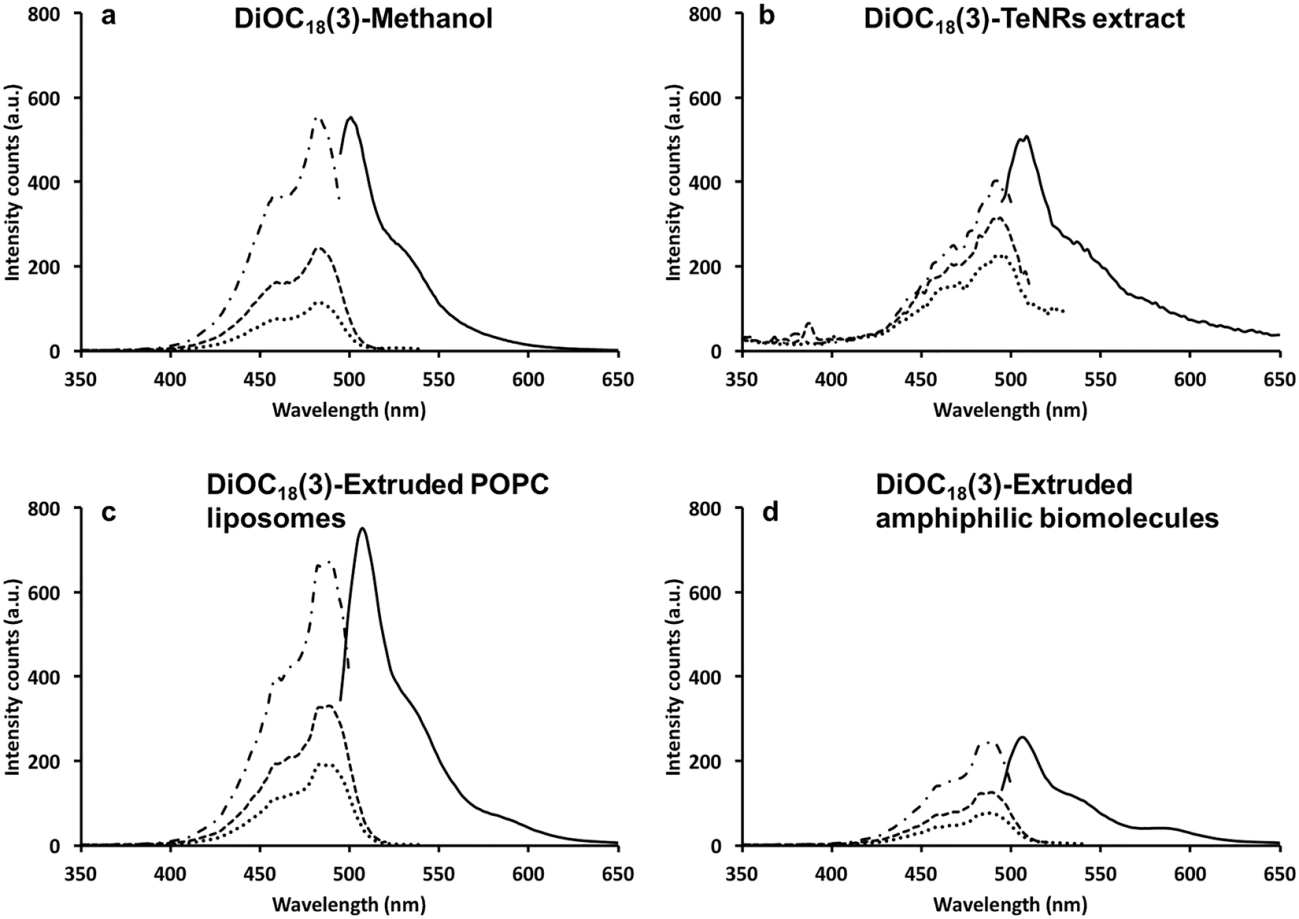
Fluorescence excitation and emission spectra. The Lipophilic tracer DiOC_18_(3) was utilized to collect the emission (λ 484 nm) and excitation ( λ 500 nm;  λ 530 nm; ∙ ∙ ∙ ∙ λ 550 nm) fluorescent spectra when it was dissolved in methanol (**a**), in association with the biogenic TeNRs extract (**b**) or extruded POPC liposomes (**c**) or the isolated and extruded amphiphilic molecules (**d**).

Chemical synthesis of TeNRs is mostly reliant on the addition to the reaction system of surfactant molecules^32–34^ that adsorb onto the TeNP surface limiting their aggregation^31^. Once the transition from *a*-Te to *t*-Te takes place, the formation of single-crystalline NRs is driven by the surfactant molecules that strongly interact with Te^0^ atoms, confining the growth of TeNRs along one axis without affecting their diameter^30,31,35,36^. Since little variation in the diameter of the biogenic TeNRs was detected (Supplementary Table S2), this observation indicated that amphiphilic biomolecules supplied by the BCP1 cells acting as surfactants could mediate the growth of NRs along one axis. To evaluate this, the existence of amphiphilic molecules within the aqueous TeNRs extract was here assessed exploiting the lipophilic tracer DiOC_18_(3) capable of specifically binding to the hydrophobic moieties of amphiphilic molecules in the extract, leading to a change in fluorescence^37^, as the unbound tracer molecules are quenched in water^38^. Indeed, a fluorescent emission peak at 507 nm was detected for the TeNRs extract (DiOC_18_(3)-TeNRs extract), which was comparable to that of the lipophilic tracer dissolved in methanol (DiOC_18_(3)-methanol; 501 nm) (Fig. 6(a,b)). These results were confirmed by the excitation spectra, which showed the same peaks for DiOC_18_(3)-TeNRs extract and DiOC_18_(3)-methanol (Fig. 6(a,b)). To understand whether the amphiphilic biomolecules detected within the TeNRs extract could chemically resemble surfactants, these macromolecules were isolated and extruded and their behaviour was compared to that of extruded POPC liposomes. Firstly, DLS analyses revealed similar size distributions of the POPC liposomes (105 ± 4.7 nm; PdI = 0.146) and the amphiphilic biomolecules (105 ± 6.4 nm; PdI = 0.166) (Supplementary Fig. S9), indicating their capability to auto-assemble at the nanoscale in aqueous solution. Moreover, the amphiphilic biomolecules and POPC liposomes labelled with DiOC_18_(3) showed emission and excitation fluorescence peaks at the same wavelengths compared to those of DiOC_18_(3)-TeNRs extract (Fig. 6c and d). Additionally, fluorescence correlation spectroscopy (FCS) was performed to evaluate variations in the diffusion times and coefficients of the lipophilic tracer when it was added to (i) TeNRs extract, (ii) POPC liposomes, and (iii) the isolated amphiphilic biomolecules (Table 1). These results showed a higher diffusion coefficient of DiOC_18_(3)-methanol as compared to those obtained for both DiOC_18_(3)-TeNRs extract and DiOC_18_(3)-extruded amphiphilic biomolecules, which were similar to that of DiOC_18_(3)-extruded POPC liposomes (Table 1).

**Table 1:** DiOC_18_(3) diffusion times and coefficients evaluated by FCS.

Samples	Diffusion coefficient (μm^2^ s^−1^)	Diffusion time (ms)
DiOC_18_(3)-methanol	345 ± 50	0.22 ± 0.08
DiOC_18_(3)-extruded amphiphilic biomolecules	4.78 ± 1.37	15.7 ± 1.62
DiOC_18_(3)-extruded POPC liposomes	4.20 ± 1.11	17.9 ± 2.42
DiOC_18_(3)-TeNRs extract	3.79 ± 1.86	19.8 ± 3.47

**Figure 7:**
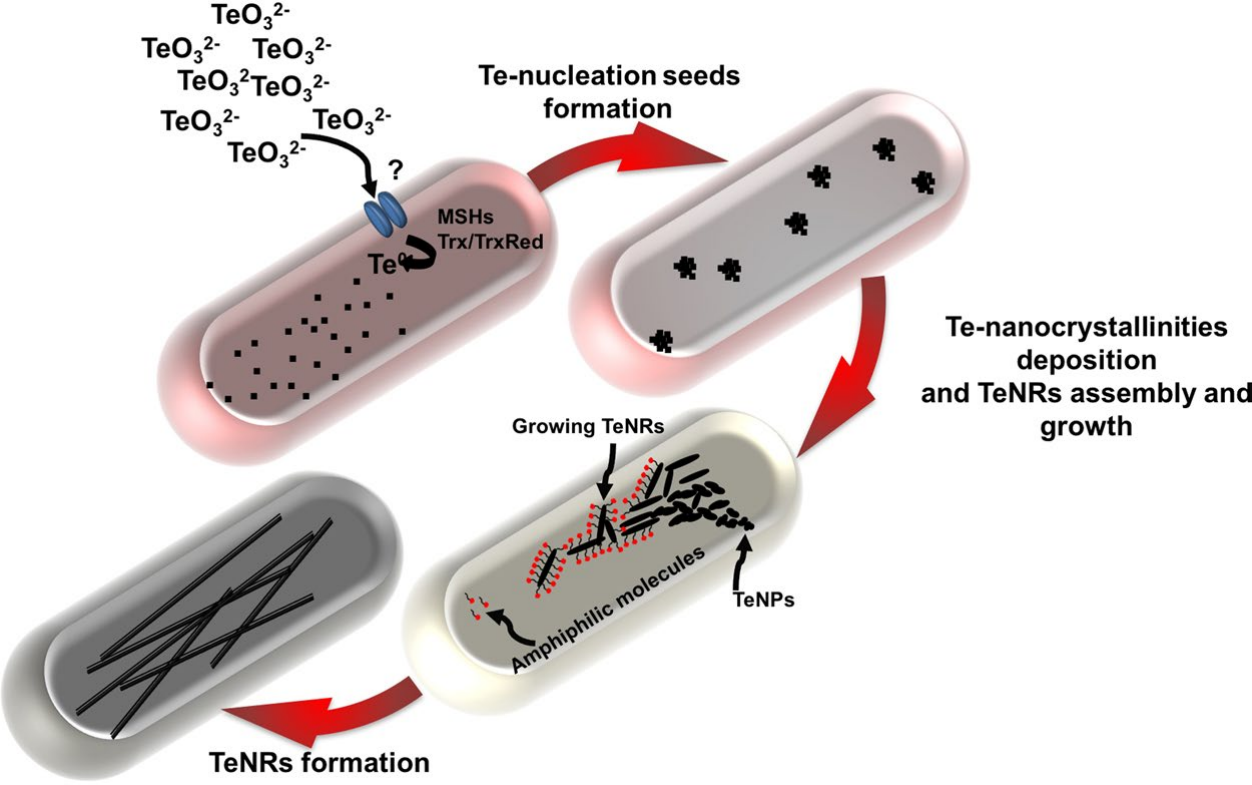
Intracellular assembly and growth of biogenic TeNRs. Once internalized by BCP1 cells, TeO_3_^2−^ bioconversion leads to the formation of Te^0^ atoms, whose concentration increases as the BCP1 exposure time to the oxyanions increases. Over the incubation time, Te^0^ atoms reach such a critical intracellular concentration that determines their aggregation, counteracting their thermodynamic instability within the cellular environment. This event results in the formation of Te nucleation seeds, whose intracellular concentration increases the more is the extent of TeO_3_^2−^ bioconversion. Te-seeds then collapse each other forming TeNPs featured by *a*-Te, which is less thermodynamic stable compared to *t*-Te. In this respect, biogenic TeNPs tend to dissolve providing Te^0^ atoms that deposit as *t*-Te nanocrystallinities, which then grow intracellularly along one axis forming TeNRs, whose growth process might be assisted by the amphiphilic molecules co-produced by the BCP1 strain.

Thus, the fluorescence spectra and the FCS analyses strongly suggested that the amphiphilic biomolecules present within the aqueous biogenic TeNRs extract behaved as non-ionic surfactants (POPC), which have been previously utilized as driving force and stabilizers for chemically synthesized NRs^39,40^. Based on these evidences, a mechanism explaining the assembly and growth of TeNRs occurring within BCP1 resting cells is proposed in Fig. 7.

### Electrical conductivity of the biogenic TeNRs extract

Since Te is a well-known narrow band-gap *p*-type semiconductor^41^, it exhibits high photoconductivity, piezoelectricity, thermoelectricity and non-linear optical response properties^42,43^, allowing the use of Te-based nanomaterials as optoelectronic, thermoelectric, piezoelectric devices, as well as gas sensors and infrared detectors^44–48^. Thus, we explored the conductive properties of the biogenic TeNRs extract, measuring its resistance (R) through the Four Probe technique^49^. The biogenic TeNRs extract air dried on the silicon support gave a low resistance value (R = 8 ± 1 Ω), compared to that of the silicon chip itself (R = 281 ± 7 Ω), and the isolated amphiphilic molecules (R = 145 ± 2 Ω). These values corresponded to an electrical conductivity (σ) of 3.0 ± 0.5, 0.08 ± 0.002 and 0.16 ± 0.02S m^−1^, respectively. Hence, TeNRs within the extract were shown to be electrically conductive, approaching the electrical conductivity values of those chemically synthesized, with σ ranging between 8 and 10S m^−1^ ^50,51^.

## Conclusions

The present study highlights the capability of the strictly aerobic *Rhodococcus aetherivorans* BCP1 strain to tolerate very high concentrations of the toxic oxyanion TeO_3_^2−^ under the physiological state of resting cells as compared to those actively growing^21^. Although the biotic conversion of TeO_3_^2−^ led to the intracellular production of different tellurium nanomaterial morphologies at early time points, the main nanostructure biosynthesized was TeNRs, whose average length was impressively long as compared to TeNRs reported in the literature so far. Moreover, the biogenic TeNRs showed a single-crystalline structure resembling those chemically synthesized, while the morphological changes of biogenic Te-nanostructures and the unchanged average diameter of the TeNRs suggested a specific mechanism of their assembly and confined growth within BCP1 cells, which might be assisted by the co-produced amphiphilic biomolecules from this *Rhodococcus* strain. Finally, the biogenic TeNRs extract showed to be electrically conductive, approaching those chemically produced and, therefore, underlining the suitability of this strain as an eco-friendly cell factory exploitable to synthesize valuable Te-based nanomaterials for future technological uses.

## Methods

### Bacterial strain and exposure conditions

*Rhodococcus aetherivorans* BCP1 strain (DSM 44980) was cultured as described elsewhere^21^, whose details are indicated in the Supplementary Information. The number of viable cells is reported as average of the Colony Forming Unit (log_10_[CFU mL^−1^]) for each biological trial (n = 3) with standard deviation (SD). All the reagents were purchased from Sigma-Aldrich^®^.

### TeO_3_^2−^ removal assay

The extent of TeO_3_^2−^ removal by BCP1 resting cells during the exposure timeframe was estimated as published elsewhere^52^ and described in detail in the Supplementary Information. The data are reported as average (n = 3) of the percentage value corresponding to TeO_3_^2−^ removal over the incubation time with SD. Further, since any statistical difference was observed between the CFU mL^−1^ counted at the earliest stages of BCP1 resting cells incubation to each oxyanion concentration tested, the specific rate of TeO_3_^2−^ bioconversion (expressed as μg mL^−1^ h^−1^) was calculated using a linear regression of the data collected over 3 h.

### Recovery of Te-nanostructure extracts and electron microscopy imaging

Te-nanostructure extracts were recovered from BCP1 resting cells following the procedure published in our previous study^21^, while Transmission Electron Microscopy (TEM) imaging of both TeO_3_^2−^-exposed BCP1 resting cells and Te-nanostructure extracts was performed using a Hitachi H7650 TEM. For bright field (BF) and high-resolution (HR) TEM, as well as the corresponding Selected-Area Electron Diffraction (SAED) pattern of TeNRs were collected by FEI Tecnai F20 TEM at an acceleration voltage of 200 kV. The samples were prepared by mounting 5 µL of either cellular suspensions or Te-nanostructure extracts on carbon-coated copper grids (CF300-CU, Electron Microscopy Sciences), which were air-dried prior the imaging. TEM micrographs were analyzed through ImageJ software to measure the actual length of TeNRs, which was calculated considering 100 randomly chosen nanorods. The distribution was fitted to a Gaussian function to yield the average length of TeNRs.

### Sample preparation

For experiments aimed to evaluate the presence of amphiphilic biomolecules within the TeNRs extract, the sample containing the amphiphilic biomolecules without biogenic nanomaterial was isolated as described elsewhere^21^. As a control experiments, a liposome solution (5 mM) was prepared by dissolving in 5 mL of water 19 mg of 1-palmitoyl-2-oleoyl-*sn*-glycero-3-phosphocholine (POPC) (Avanti^®^ Polar Lipids, Inc), which is non-ionic surfactant^53^. The POPC liposome solution and the isolated amphiphilic biomolecules were extruded through a Mini-Extruder equipped with a polycarbonate membrane (0.1 μm) (Avanti^®^ Polar Lipids, Inc).

### Dynamic Light Scattering (DLS)

DLS measurements were performed onto 1 mL of (i) the isolated and extruded amphiphilic biomolecules and (ii) the POPC liposome solution by using Zen 3600 Zetasizer Nano ZS™ from Malvern Instruments.

### Fluorescence spectroscopy and Fluorescence Correlation Spectroscopy (FCS) analyses

The lipophilic tracer 3,3′-dioctadecyloxacarbocyanine perchlorate (DiOC_18_(3) Invitrogen™) dissolved in methanol was used as the probe in all experiments. 3 mL of DiOC_18_(3) stock solution (0.4 mM) was utilized to obtain fluorescence spectra of the free dye. TeNRs extract, as well as the isolated and extruded amphiphilic molecules were incubated with the dye previously dried under argon flow, incubating 3 mL of each sample for 30 minutes at room temperature with shaking. Further, the POPC liposome solution was dried along with the dye under argon flow, then resuspended in 3 mL of water to incorporate the lipophilic tracer within the liposomes. The samples were excited (λ_ex_) at 488 nm and fluorescence emission spectra were collected above 495 nm wavelength using a Varian *Cary Eclipse* fluorescence spectrophotometer, while excitation spectra were obtained for 3 different fixed wavelengths (λ_fix_; 510, 530 and 550 nm).

FCS experiments were carried out with an ISS Alba IV Confocal Spectroscopy & Imaging Workstation coupled with a Nikon Eclipse Ti-U microscope. The lipophilic tracer was diluted to a final concentration of 2 nM, and 400 μl of this dilution was used to perform FCS. The autocorrelation curves corresponding to all the samples were obtained from 15 independent runs by exiting the dye with a single photon CW Ar-laser (λ_ex_ = 488 nm). All the autocorrelation functions were built by the Vistavision ISS software and fitted according to a theoretical model for three-dimensional (3D) global diffusion, assuming that the detection volume was approximated by a 3D Gaussian function^54^. Based on the fitted autocorrelation functions, for each sample the diffusion coefficient and the time of diffusion were calculated.

### Four Probe technique

The electrical property of the TeNRs extract was studied by air drying 800 μL of sample onto a 2 × 1 cm Crystal Silicon wafer (type N/Phos, size 100 mm, University Wafer), whose contacts were drawn through silver painting (PELCO^®^ TED PELLA, INC.) to enhance the resistance values recorder by 5492B Digit Multimeter (BK PRECISION^®^). The obtained resistance values correspond to the average of 6 independent measurements with SD.

### Data availability

All data generated or analysed during this study are included in this published article (and its Supplementary Information files).

## Electronic supplementary material

Supplementary Information
