# Supplementary material for: Assembly, growth and conductive properties of tellurium nanorods produced by Rhodococcus aetherivorans BCP1
Source: Sci Rep. 2018 Mar 2;8:3923. doi: 10.1038/s41598-018-22320-x (PMC5834534; doi:10.1038/s41598-018-22320-x)
Supplement: Supplementary file 1 — Supplementary Information [file 41598_2018_22320_MOESM1_ESM.doc]

Supplementary information for:

**Assembly, growth and conductive properties of tellurium nanorods produced by *Rhodococcus aetherivorans* BCP1**

Alessandro Presentato^1,*^, Elena Piacenza^1^, Ali Darbandi^2^, Max Anikovskiy^3^, Martina Cappelletti^4^, Davide Zannoni^4^, Raymond J. Turner^1,*^

^1^*Microbial Biochemistry Laboratory, Department of Biological Sciences, University of Calgary, 2500 University Dr. NW, Calgary, AB T2N 1N4, Canada*

*^2^Microscopy and Imaging Facility, Cumming School of Medicine, University of Calgary, 3330 Hospital Dr. NW, Calgary, AB T2N 4N1, Canada*

^3^*Department of Chemistry, University of Calgary, 2500 University Dr. NW, Calgary, AB T2N 1N4, Canada*

^4^*Unit of General and Applied Microbiology, Department of Pharmacy and Biotechnology, University of Bologna, Via Irnerio 42, Bologna, 40126, Italy*

^*^*Corresponding authors*

***Contact information:***

*Alessandro Presentato: alessandro.presentat@ucalgary.ca*

*Elena Piacenza: elena.piacenza@ucalgary.ca*

*Ali Darbandi: ali.darbandi@ucalgary.ca*

*Max Anikovskiy: m.anikovskiy@ucalgary.ca*

*Martina Cappelletti: martina.cappelletti2@unibo.it*

*Davide Zannoni: davide.zannoni@unibo.it*

*Raymond J. Turner: turnerr@ucalgary.ca*

**Supplementary Methods**

**Bacterial strain, growth media, exposure conditions.** BCP1 biomass was pre-cultured in 250 mL Erlenmeyer Baffled Flask for 48 h containing 25 mL of Luria-Bertani medium (indicated as LB) [composition (g L^-1^) NaCl, 10; Yeast Extract, 5; Tryptone, 10]. When necessary the medium was solidified by adding 15 g L^-1^ of Agar. BCP1 cells were then inoculated (1% v/v) and grown for further 48 h in 50 mL of LB broth. After this incubation step, BCP1 LB-grown cells were collected by centrifugation (3,700 rpm) for 10 minutes and washed twice with 50 mL of Phosphate-buffered Saline (PBS) [containing (g L^-1^) NaCl, 8; KCl, 0.2; Na_2_HPO_4_, 1.44; KH_2_PO_4_, 0.24] pH 7.4. The obtained resting cells were exposed for 0.5, 1, 3, 6 and 16 h in 50 mL of PBS supplied with 100, 500 or 1000 μg mL^-1^, as initial concentration, of K_2_TeO_3_.

All TeO_3_^2-^ exposure experiments with BCP1 cells were performed at 30°C with shaking (150 rpm).

The survival extent of BCP1 TeO_3_^2-^-exposed cells was evaluated by spot plate count method, recovering the spotted cells onto LB agar plates for 48 h at 30°C.

**TeO_3_^2-^ bioconversion assay.** Briefly, 1 mL of BCP1 resting cells exposed to different concentrations of K_2_TeO_3_ was collected for each time point considered. The sample was centrifuged at 14,000 rpm for 2 minutes to separate the bacterial cell pellet from the supernatant. A 10- to 100 µL aliquot of supernatant was mixed with 600 µL of 0.5 M Tris-HCl buffer pH 7.0 (VWR®), 200 µL of diethyldithiocarbamate (Sigma-Aldrich®), and PBS up to a final volume of 1 mL. The absorbance of the mixture was read at 340 nm using a Varian Cary® 50 Bio UV-Visible Spectrophotometer. The abiotic removal of TeO_3_^2-^ was also evaluated in the case of two different control experiments, which consisted in the incubation of the highest amount of TeO_3_^2-^ tested (1000 μg mL^-1^) in PBS only and/or containing autoclaved biomass. The residual concentration of TeO_3_^2-^ was determined using the absorbance values obtained from each experimental condition, which were fitted to the calibration curve derived from the analysis of known K_2_TeO_3_ concentrations (0, 10, 20, 30, 40 and 50 μg mL^-1^) in PBS (R^2^=0.98).

The highest TeO_3_^2-^ concentration (1000 μg mL^-1^) tested in this study has been incubated at 30°C with shaking over a specific timeframe (0, 0.5, 1, 3, 6 and 16 h) either in Phosphate Buffer Saline (PBS) or PBS containing autoclaved biomass (sterile control), to evaluate whether during the exposure time an abiotic loss of the oxyanion supplied to BCP1 resting cells occurred.


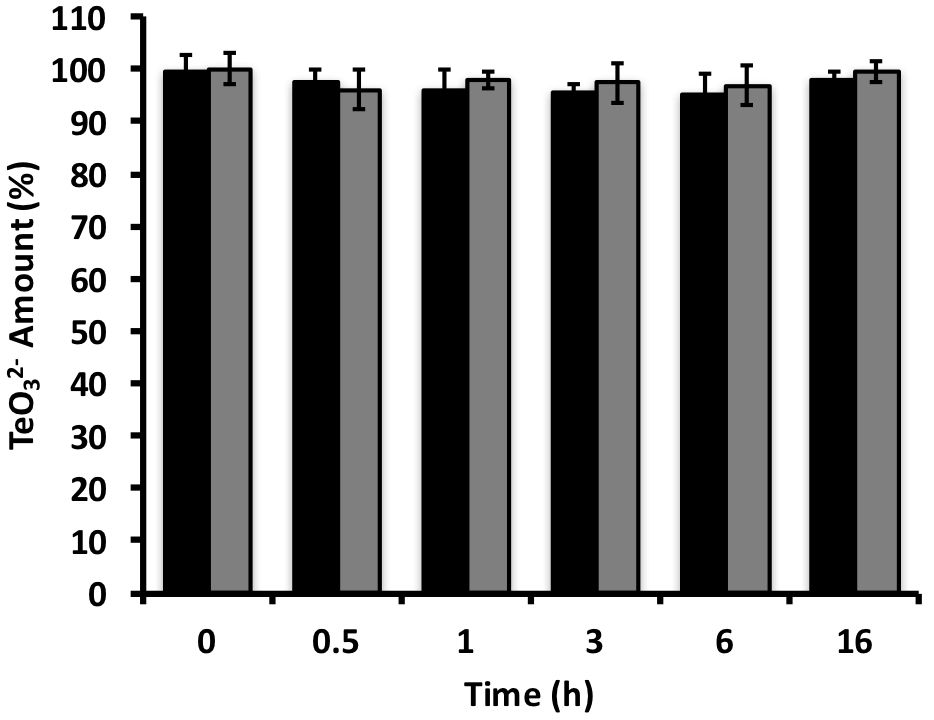


**Supplementary Figure 1 - Abiotic control experiments.** Evaluation of TeO_3_^2-^ removal when it was supplied to PBS (
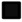
) or PBS containing autoclaved biomass (
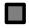
) over the incubation time. The error bars indicate the standard deviation three biological replicates.

The Supplementary Figures 2, 3 and 4 show the complete time course of formation and growth of Te-nanostructures, which occurred within BCP1 resting cells as function of either the initial oxyanion concentration tested (100, 500 and 1000 μg mL^-1^) or the cell exposure time (0, 0.5, 1, 3, 6 and 16 h).


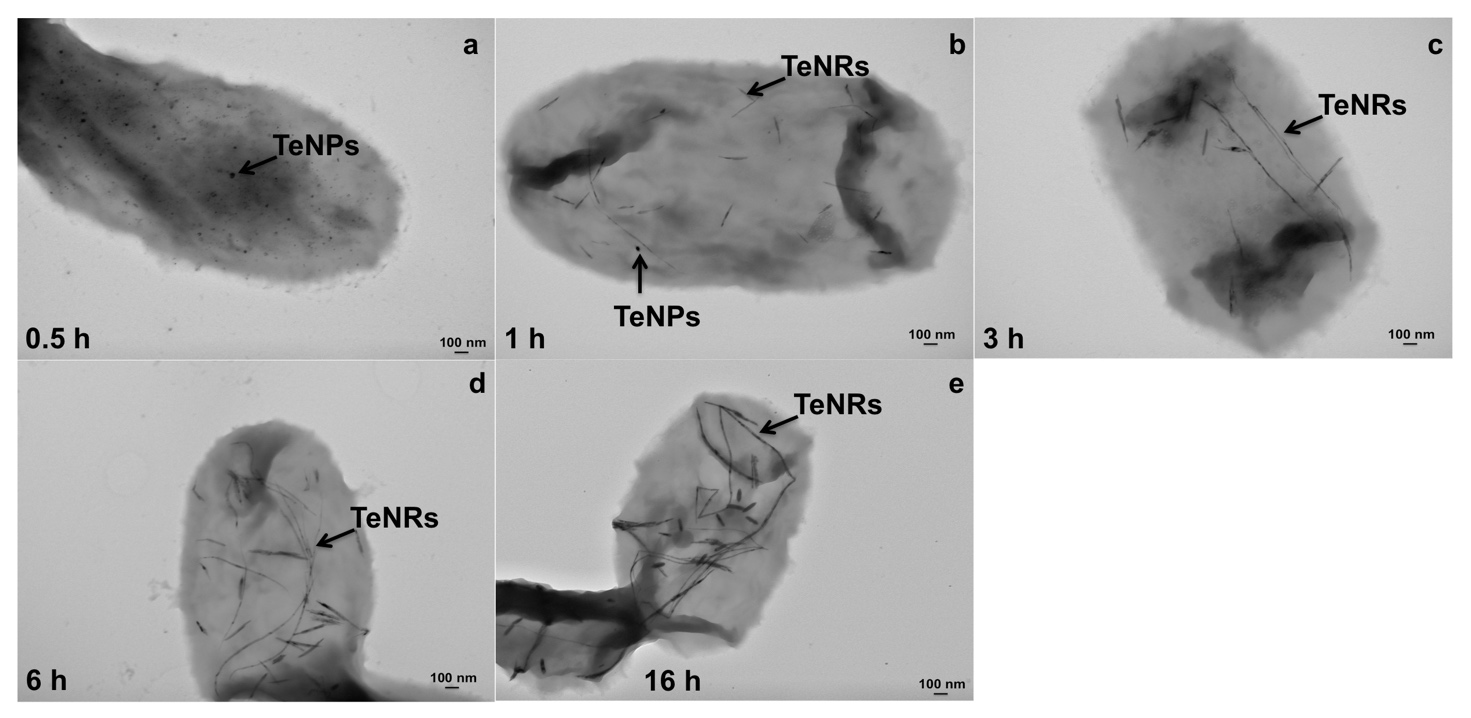


**Supplementary Figure 2 - Transmission Electron Microscopy imaging of BCP1 resting cells exposed to 100 μg mL^-1^ of TeO_3_^2-^.** Intracellular formation of Te-nanostructures over time. The biogenic Te-nanomaterial in the form of Te-nanoparticles (TeNPs) and Te-nanorods (TeNRs) is highlighted by black arrows. Scale bar = 100 nm.


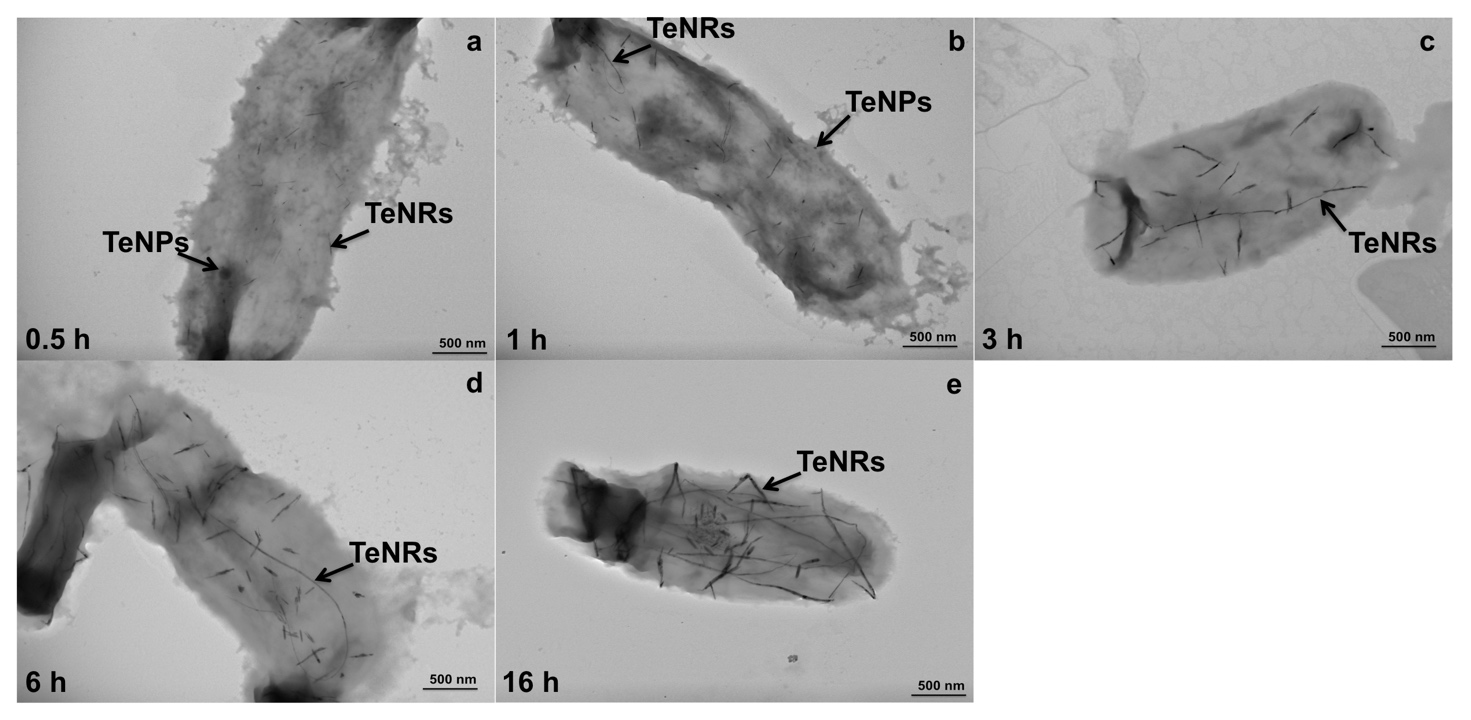


**Supplementary Figure 3 - Transmission Electron Microscopy imaging of BCP1 resting cells exposed to 500 μg mL^-1^ of TeO_3_^2-^.** Intracellular formation of Te-nanostructures over time. The biogenic Te-nanomaterial in the form of Te-nanoparticles (TeNPs) and Te-nanorods (TeNRs) is highlighted by black arrows. Scale bar = 500 nm.


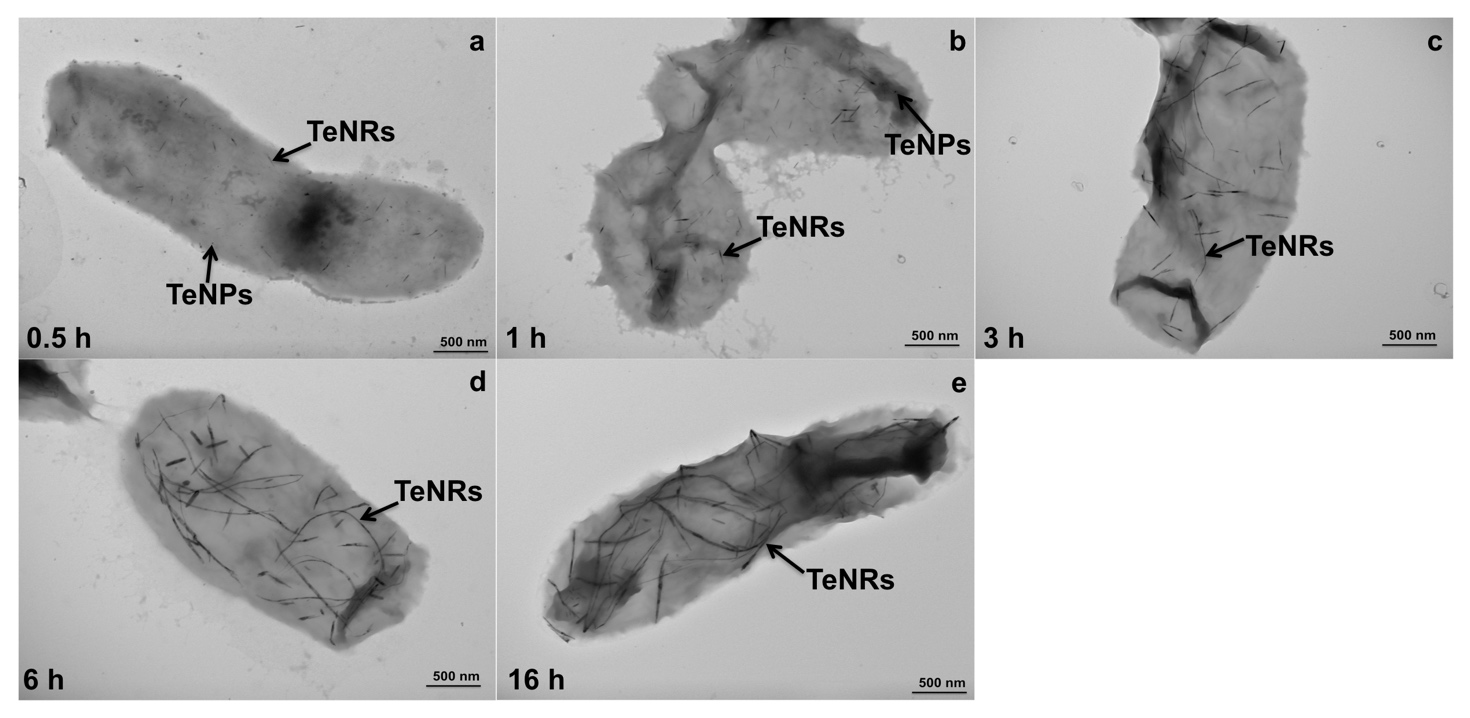


**Supplementary Figure 4 - Transmission Electron Microscopy imaging of BCP1 resting cells exposed to 1000 μg mL^-1^ of TeO_3_^2-^.** Intracellular formation of Te-nanostructures over time. The biogenic Te-nanomaterial in the form of Te-nanoparticles (TeNPs) and Te-nanorods (TeNRs) is highlighted by black arrows. Scale bar = 500 nm.

The Supplementary Figures 5, 6 and 7 show a time course experiment carried out on the recovered biogenic Te-nanomaterial extracts from BCP1 resting cells exposed to the different concentration of TeO_3_^2-^ tested for different times, in order to evaluate changes in the nanomorphology of Te-nanostructures as function of both TeO_3_^2-^ concentration and cell exposure time.


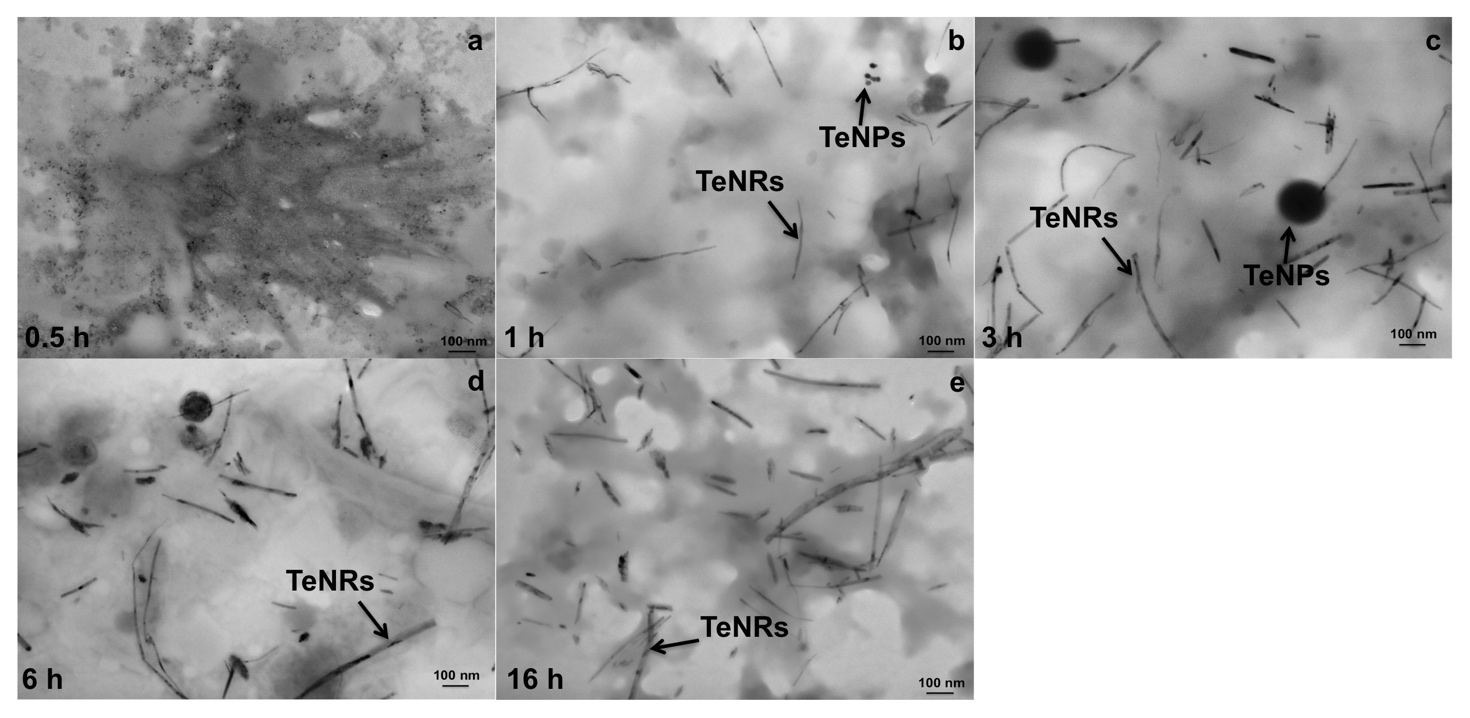


**Supplementary Figure 5 - Transmission Electron Microscopy imaging of Te-nanostructure extracts generated by BCP1 resting cells exposed to 100 μg mL^-1^ of TeO_3_^2-^.** Electron micrographs of the biogenic Te-nanomaterial recovered from BCP1 cells over the exposure time. Te-nanomaterial in the form of Te-nanoparticles (TeNPs) and Te-nanorods (TeNRs) is highlighted by black arrows. Scale bar = 100 nm.


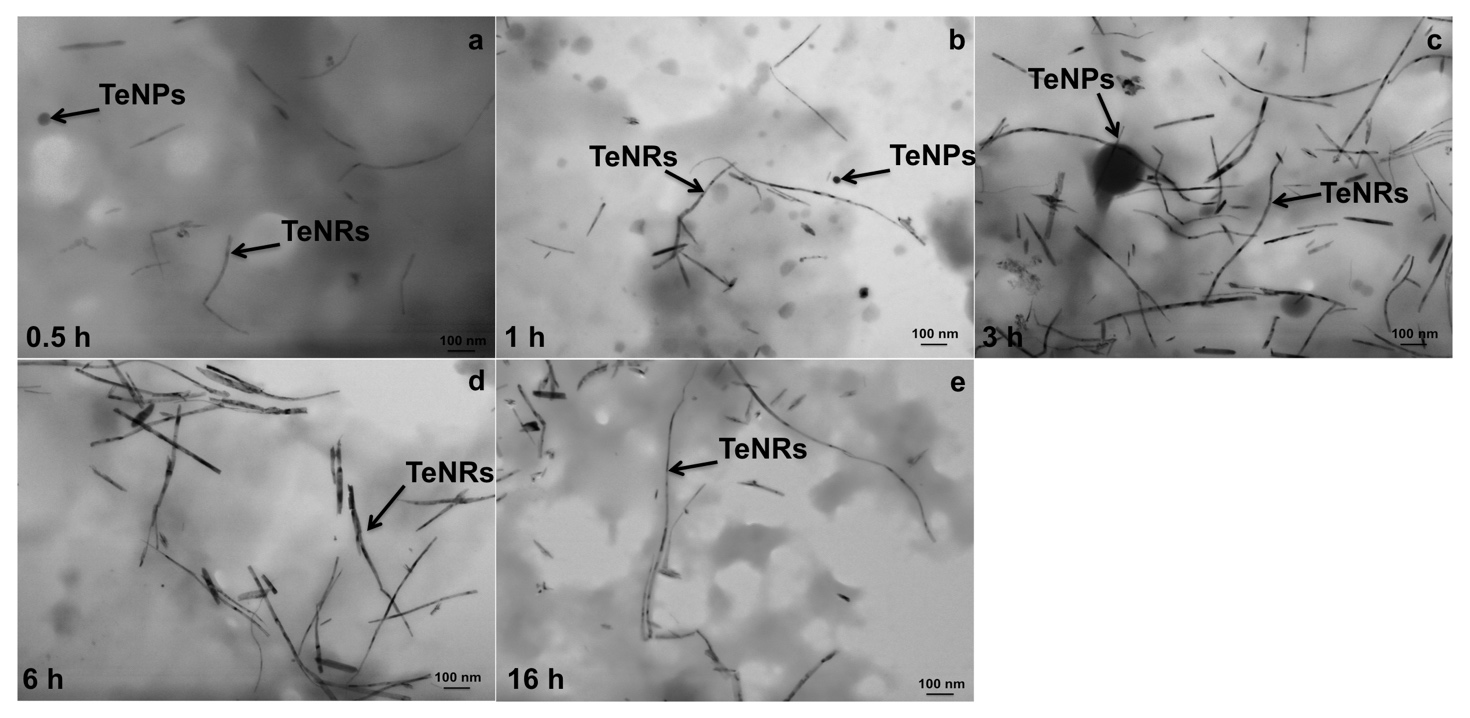


**Supplementary Figure 6 - Transmission Electron Microscopy imaging of Te-nanostructure extracts generated by BCP1 resting cells exposed to 500 μg mL^-1^ of TeO_3_^2-^.** Electron micrographs of the biogenic Te-nanomaterial recovered from BCP1 cells over the exposure time. Te-nanomaterial in the form of Te-nanoparticles (TeNPs) and Te-nanorods (TeNRs) is highlighted by black arrows. Scale bar = 100 nm.


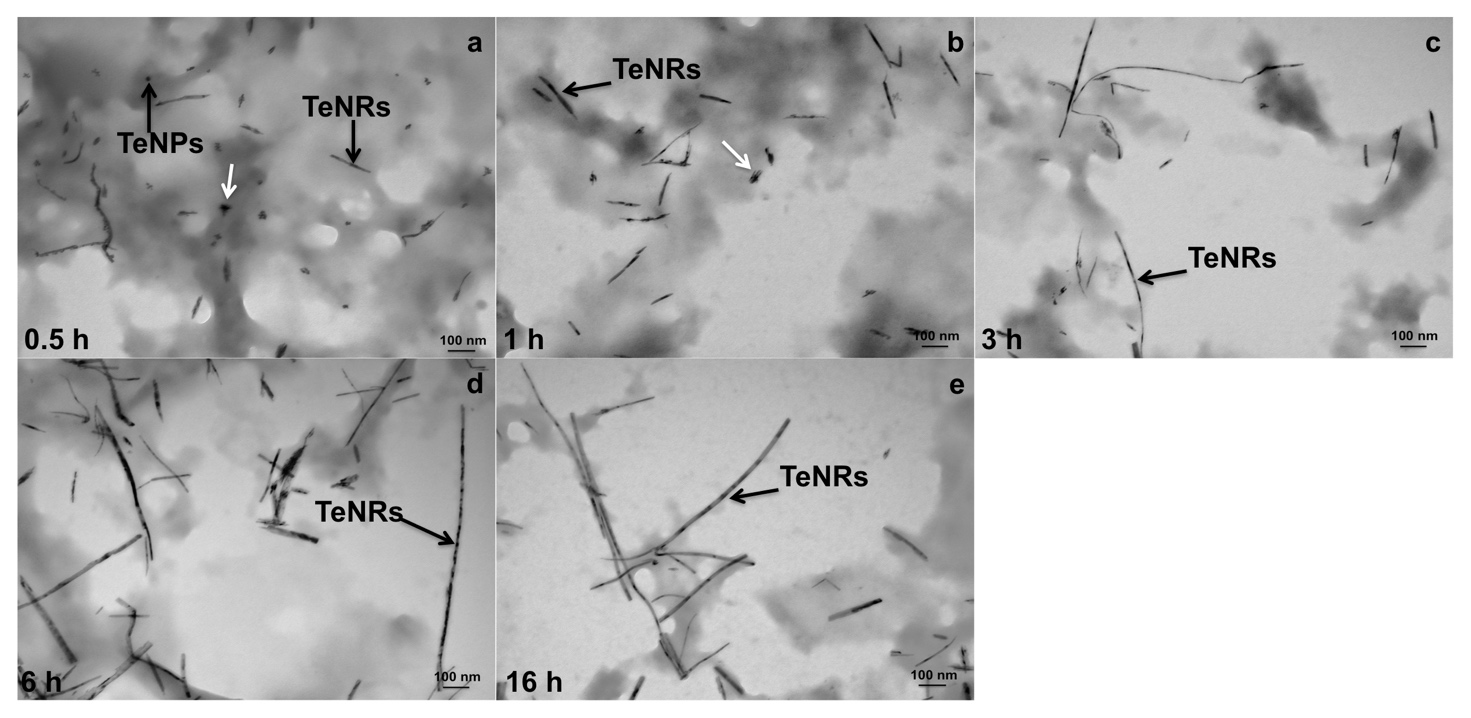


**Supplementary Figure 7 - Transmission Electron Microscopy imaging of Te-nanostructure extracts generated by BCP1 resting cells exposed to 1000 μg mL^-1^ of TeO_3_^2-^.** Electron micrographs of the biogenic Te-nanomaterial recovered from BCP1 cells over the exposure time. Te-nanomaterial in the form of Te-nanoparticles (TeNPs) and Te-nanorods (TeNRs) is highlighted by black arrows, while the white arrows indicate the shard-like nanoparticles. Scale bar = 100 nm.

The dependency of Tellurium nanorods (TeNRs) average length on the TeO_3_^2-^ concentration and cell exposure time was evaluated by measuring 100 randomly chosen nanorods over several electron micrographs of TeNRs extracts recovered from BCP1 resting cells for each experimental condition tested. In the Supplementary Figure 8 is shown the evolution of the TeNRs average length, from shorter to longer ones, as function of both initial amount of TeO_3_^2-^ supplied to BCP1 resting cells and their exposure time.


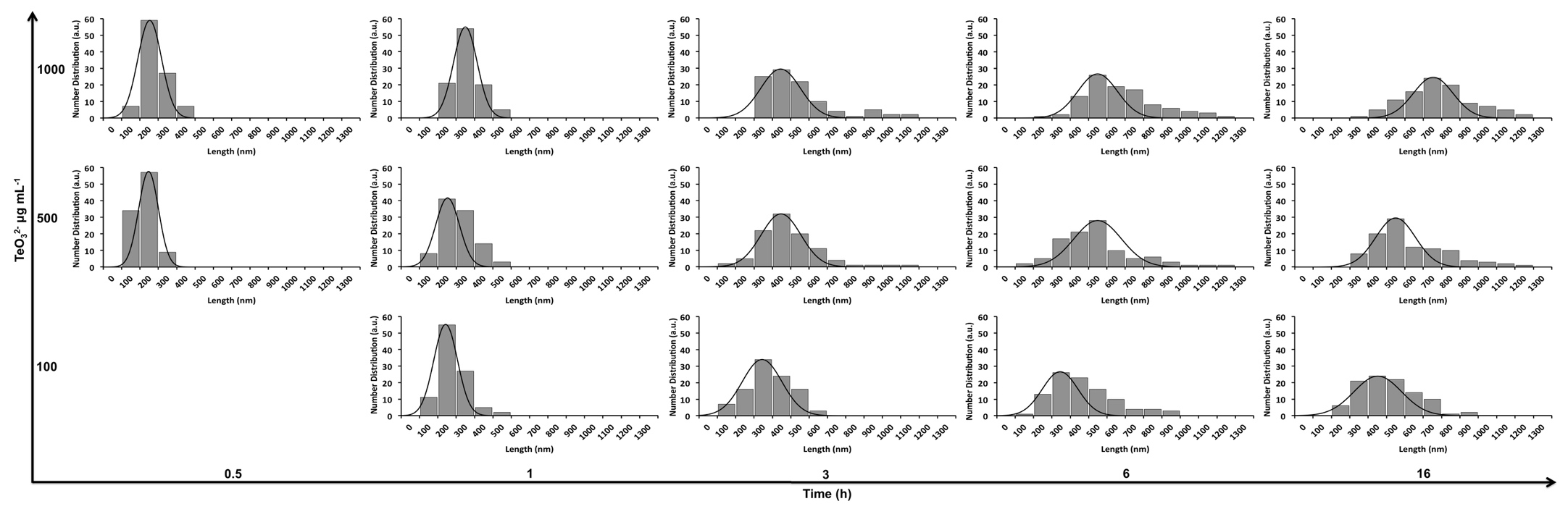


**Supplementary Figure 8 - Tellurium nanorods (TeNRs) average length distribution.** Dependency of the biogenic TeNRs average length (
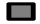
) measured on the initial TeO_3_^2-^ concentration and cell exposure time. The distribution was fitted to a Gaussian function (
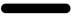
) to yield TeNRs average length.

Dynamic Light Scattering (DLS) analysis has been performed to evaluate the capability of the isolated and extruded amphiphilic molecules from the TeNRs extract to auto-assemble at the nanoscale, comparing their behavior with a solution of extruded POPC liposomes.


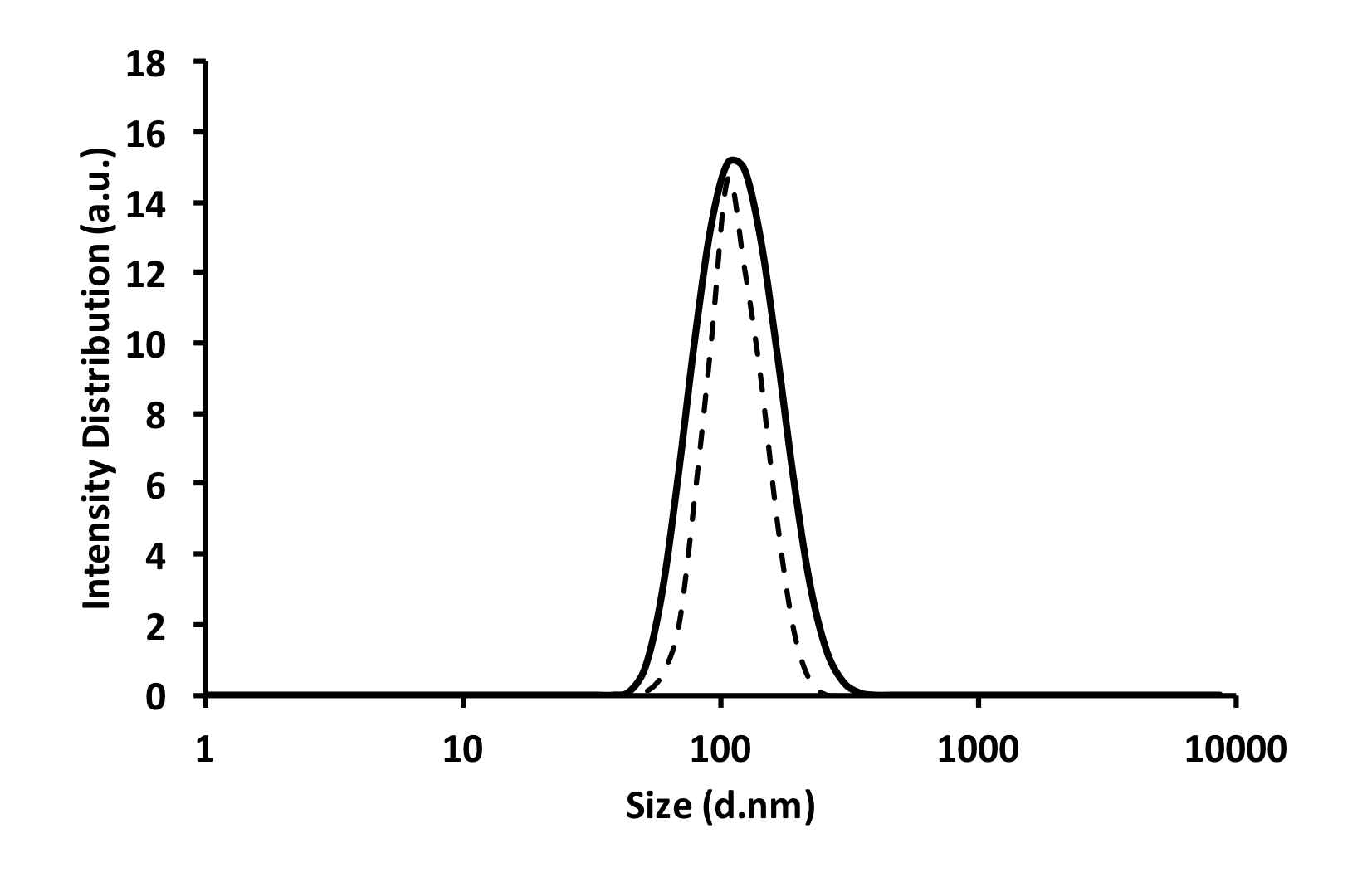


**Supplementary Figure 9 - Dynamic Light Scattering (DLS).** Size distribution of the POPC liposomes (
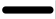
) and isolated and extruded amphiphilic molecules (
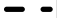
).

**Table S1: TeNRs average length (nm) produced by *Rhodococcus aetherivorans* BCP1 resting cells.**

| **TeNRs average length (nm) per initial TeO_3_^2-^ concentration [μg mL^-1^]** | | | |
| --- | --- | --- | --- |
| **Time (h)** | **100** | **500** | **1000** |
| 0.5 | N.M. | 123±49 | 185±66 |
| 1 | 181±79 | 214±92 | 260±72 |
| 3 | 388±120 | 488±174 | 539±192 |
| 6 | 468±174 | 543±201 | 677±195 |
| 16 | 509±153 | 632±201 | 781±189 |

Average length not measured is indicated as N.M.

**Table S2: TeNRs average diameter (nm) produced by *Rhodococcus aetherivorans* BCP1 resting cells.**

| **TeNRs average diameter (nm) per initial TeO_3_^2-^ concentration [μg mL^-1^]** | | | |
| --- | --- | --- | --- |
| **Time (h)** | **100** | **500** | **1000** |
| 0.5 | N.M. | 5±2 | 6±2 |
| 1 | 7±2 | 6±2 | 8±2 |
| 3 | 9±3 | 8±2 | 8±2 |
| 6 | 9±3 | 9±2 | 9±3 |
| 16 | 10±3 | 10±3 | 10±3 |

Average length not measured is indicated as N.M.
